# The Application of 3D Landmark-Based Geometric Morphometrics towards Refinement of the Piglet Grimace Scale

**DOI:** 10.3390/ani12151944

**Published:** 2022-07-30

**Authors:** Maria E. Lou, Samantha T. Porter, Jason S. Massey, Beth Ventura, John Deen, Yuzhi Li

**Affiliations:** 1 Department of Animal Science, University of Minnesota, St. Paul, MN 55108, USA; melou3@vet.k-state.edu (M.E.L.); bventura@lincoln.ac.uk (B.V.); 2 West Central Research and Outreach Center, University of Minnesota, Morris, MN 56267, USA; 3 Liberal Arts Technologies and Innovation Services, College of Liberal Arts, University of Minnesota, Minneapolis, MN 55455, USA; port0228@umn.edu; 4 Department of Integrative Biology and Physiology, Medical School, University of Minnesota, Minneapolis, MN 55455, USA; jason.massey@monash.edu; 5 Department of Veterinary Population Medicine, College of Veterinary Medicine, University of Minnesota, St. Paul, MN 55108, USA; deenx003@umn.edu

**Keywords:** castration, pain, photogrammetry, animal welfare

## Abstract

**Simple Summary:**

Piglet welfare on commercial farms is negatively affected by the routine husbandry procedure of surgical castration. Objective identification of pain is necessary to improve on-farm piglet welfare and for the development of proper pain management. The Piglet Grimace Scale (PGS) is a method of pain assessment using an ordinal scale that quantifies changes in facial expression induced by acute pain. This study evaluated the application of 3D landmark geometric morphometrics in refining existing PGS methods. To determine if these methods could objectively identify alterations in pig facial shape through landmark analysis, piglet images were taken using a multi-camera rig. Images were then used to construct 3D facial models using photogrammetry (structure from motion). Neither established nor refined PGS methods were able to detect pain-induced facial expressions. Further research is warranted to refine the PGS.

**Abstract:**

Proper methods of assessment to objectively identify pain are essential for welfare improvements of piglets undergoing surgical castration on commercial farms. The Piglet Grimace Scale (PGS) is used to identify changes in facial expressions caused by acute pain in piglets undergoing tail docking and castration. However, subjective scoring methods are a concern for the validation of the PGS. The objectives of this study were to evaluate and refine the PGS through 3D landmark geometric morphometrics. Male piglets (*n* = 88) were randomly allocated to one of two treatments: castration and sham-castration. Piglet facial images were taken at four time points (before treatment, immediately post-treatment, 1 h post-treatment, and 4 h post-treatment) using a photogrammetry rig. Images were scored by four raters using five facial action units (FAUs): orbital tightening, ear position, temporal tension, lip contraction, and nose bulge/cheek tension. Three-dimensional facial models were generated and landmarked denoting 3 FAUs (orbital tightening, lip contraction, and nose bulge/cheek tension). Results suggest that orbital tightening and ear position may be reliable FAUs for the PGS. However, neither the PGS nor 3D landmark-based geometric morphometrics were able (both *p* > 0.10) to reliably identify facial indicators of pain in piglets undergoing castration.

## 1. Introduction

The grimace scale is considered a novel method of acute pain assessment that identifies and assesses post-procedural changes in facial expression [1]. These pain-induced changes in facial expression are controlled by involuntary muscle movements within the major regions of the face: eyes, ears, mouth and jaw, and nose and cheeks [2]. The grimace scale uses a simplified version of the Facial Action Coding System, termed Facial Action Units (FAUs), which are specific to pain states and include orbital tightening, ear position, cheek tension, and nose bulging [2,3]. Following promising results from the first non-human grimace scale developed for mice (the Mouse Grimace Scale (MGS) [1]), grimace scales have been developed for other domestic animal species including rats, rabbits, horses, sheep, and cows [3,4,5,6,7]. Grimace scales have shown to be advantageous as they are non-invasive, provide prospective assessment, require minimal training and do not require advanced skills to use [2,8,9].

The Piglet Grimace Scale (PGS) was first described by Gottardo et al. [10] and has since been used to assess acute pain in piglets undergoing routine husbandry procedures including tail docking and surgical castration [11,12]. In the United States, nearly all male piglets are surgically castrated to avoid boar taint and facilitate animal management, although extensive research has shown it is painful to piglets [13,14,15,16,17]. Surgical castration certainly has large implications on animal welfare given the United States markets over 115 million pigs each year and approximately half are male [18]. However, the PGS and other grimace scales alike are challenged by several factors including but not limited to observer bias, inconsistent angles and lighting in photographs and video recordings, and difficulty in quantifying specific measurements (i.e., volume, length, and depth) with two-dimensional images [9,19]. It has been suggested that further grimace work should develop techniques to improve image quality by reducing animal movement in response to physical restraint, provide effective angles and uniform lighting sources, and use high-resolution techniques [10,11,12].

A three-dimensional optical system such as photogrammetry may better provide objective descriptions (e.g., shape) and precise measurements of facial grimace images [20,21]. Photogrammetry, also referred to as structure from motion, is a computational method for 3D reconstruction built from a series of overlapping 2D images [22]. Novel 3D techniques have gained much interest within livestock farming to improve management [22,23,24,25]. Unlike live visuals and 2D images, 3D data allow for the measurement of cross-sectional areas, volume, and depth, and are able to account for differences in color and background [23]. Studies have focused on both 2D and 3D image analysis for automatic pig weighing and assessment of animal shape [23,24,25,26,27,28]. Cappai et al. [25] applied photogrammetric processing to generate 3D spatial data to predict the wither’s height, body length, and rear trimness of bulls.

Combining photogrammetry with landmark-based geometrics morphometrics may provide an opportunity for further refinement of grimace scale imaging by providing an objective, quantitative description of shape variation [29]. Three-dimensional landmark-based geometric morphometrics (3DGM) use a set of landmarks placed onto a 3D model to annotate locations of biological significance [29]. These landmarks then provide the ability to measure animals and specific body parameters [29], as coordinates (2D or 3D) are representations of the subject’s shape and are aligned and scaled to eliminate any factors not related to the shape of interest [29]. A study on the domestic cat found significant coordinate differences associated with pain after an ovariohysterectomy using geometric morphometrics on 2D images [30]. However, to our knowledge, 3DGM has yet to be used to examine facial changes in pigs.

Therefore, this study aims to improve the assessment of pain in castrated piglets by refining the subjective PGS through the application of photogrammetry and 3DGM. Our primary and secondary objectives of this study were to evaluate the utility of the PGS to detect acute pain and to refine the current PGS through application of photogrammetry and 3DGM. To refine the PGS, we evaluated whether photogrammetry and 3DGM can be used to quantify changes in piglet facial shape caused by surgical castration.

## 2. Materials and Methods

### 2.1. Animals and Facilities

The animal trial was conducted at the University of Minnesota’s West Central Research and Outreach Center (WCROC) in Morris, MN in May 2019. Male piglets (*n* = 88; Yorkshire × Landrace × Duroc) born to 16 sows in a confinement farrowing barn within a week were used for this study. Sows and their litters were housed in 16 individual farrowing stalls (150 cm × 210 cm) on slatted floors. Each farrowing stall was equipped with two creep areas (45 cm × 210 cm/area) with a supplemental heat lamp in one creep area for piglets, a sow feeder, and two nipple drinkers. All sows farrowed naturally without artificial induction. Piglets were not cross-fostered or fully processed (i.e., tail docked, needle-teeth clipped, and iron injected) until completion of data collection at 48 h after birth.

Birthweights were recorded and piglets were marked with a number (1 to 10) using a Paintstik Livestock Marker (LA-CO Industries, INC., Elk Grove Village, IL, USA) within 24 h after birth for identification. Sows and their piglets remained together in their farrowing stalls until weaning at 21 days after farrowing. Room temperature in the farrowing barn was controlled at 20 °C within the thermoneutral zone for lactating sows as much as possible by thermostats that operated exhaust fans and heaters. The light period was set at 8 h daily starting from 07:00 h.

### 2.2. Experimental Treatment and Design

The experimental treatment was surgical castration without the use of analgesia (C) vs. sham-castration (S), as described previously [31]. A generalized randomized block design was employed, with litter serving as block. Within each litter, an even number of healthy male piglets were selected for the study based on visual assessment. Piglets were assigned to each treatment group using an alternate sorting method to balance the order of treatment administration within and among litters. This method of randomization consisted of litters being assigned a random number generated by Microsoft Excel (Version 16.27; Microsoft Corporation, Washington, DC, USA), where an even and odd number specified whether the first piglet within litter would be castrated or sham-castrated, respectively. Treatment assignment within each litter was alternated thereafter. Both treatments were equally represented in each litter, with a range between one to five male piglets/treatment/litter.

#### Treatment Procedures

Castration or sham-castration was performed on piglets within 24 h after birth. Identical handling and iodine disinfection procedures were followed for both treatment groups, except sham-castrated piglets were not castrated. Piglets (avg. birth weight = 1.72 kg ± 0.78 SD) were restrained and castrated or sham-castrated by the same two trained personnel each day between 08:00 and 12:00 h. On treatment day, selected male piglets were placed in a cart and moved from the farrowing room into the farrowing barn lobby. Following the WCROC’s standard on-farm procedures, piglets were restrained by their hind legs and held upside down. For castration, a scalpel incised the scrotum to externalize the testes with two vertical incisions, the spermatic cord was cut, and iodine was applied to the surgical site. Sham-castration was conducted using the dull end of the scalpel to mimic the motion of incision and testicles were manipulated over the skin to simulate removal of testicles followed by iodine application.

### 2.3. Image Collection for Photogrammetry

Photographs of piglets were taken at four time points: immediately pre-castration (T1), immediately post-castration (T2), 4 h post-castration (T3), and 22 h post-castration (T4) as in previous work [10,11,12]. To maintain a consistent piglet position and orientation for photographs, piglets were placed into a custom-built cradle (29.2 cm × 2.5 cm × 17.8 cm height), specifically designed for this study. Piglets remained in the cradle at each time point for a maximum of 20 s until a still image of the piglet was captured.

**Figure 1:**
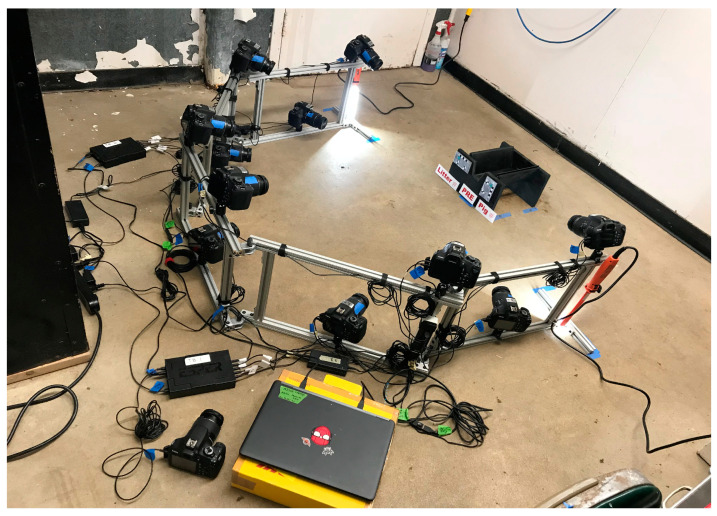
Photogrammetry rig and piglet cradle.

For 3D reconstruction of piglet models, a pilot test was conducted using a frozen (previously deceased) piglet to determine the minimum number of cameras needed to capture pig facial images from all required angles. The pilot test determined a total of 11 cameras would be sufficient. Thus, a photogrammetry rig (119.4 cm × 116.8 cm) was built to install 11 cameras (Canon EOS Rebel T5, Canon, Tokyo, Japan) to take photographs of the piglets (Figure 1). The photogrammetry rig was constructed from metal t-slot framing and fittings (McMaster-Carr, Elmhurst, IL, USA) and fitted with two ESPER TriggerBoxes (ESPER LTD, Nottingham, UK), connecting all cameras to a laptop (Hewlett-Packard, Palo Alto, CA, USA). This system made it possible to remotely trigger all 11 cameras to capture a photograph instantaneously. The rig was also equipped with two 400 L LED task lights (Southwire, Carrollton, GA, USA) for uniform lighting, as the flash was shut off to minimize disturbance of piglets. Smart Shooter (Version 3.28; Kuvacode Oy, Kerava, Finland) was installed on the laptop to control all camera settings for photo quality (JPEG Fine Large), aperture (6.3), shutter speed (1/125), and ISO (3200). For consistency, all camera lenses were set to auto focus and to maximum zoom (55 mm). The ground was marked to secure the location of the piglet cradle, which was placed at the center (7.62 cm from center of rig) of the photogrammetry rig with a similar distance to each camera for the entire study. Photos were transferred from the laptop to an external hard drive (Western Digital, San Jose, CA, USA) for further processing (i.e., PGS scoring and 3D models).

### 2.4. Image Processing and 3D Landmark-Based Geometric Morphometrics

#### Model Generation and Landmarking

The set of 2D images taken using the 11 cameras at each time point for each piglet was used to generate 3D models of piglets using the Agisoft Metashape software (Version 1.6.2; Agisoft LLC, St. Petersburg, Russia). Models were processed using the settings described in Table S1. In cases where Metashape could not align the leftmost and rightmost images and models were generated using only 9 photos (rather than 11 photos) per piglet per timepoint. Models were scaled using the ‘detect markers’ feature in Metashape on two calibrated 5 cm scale bars (Cultural Heritage Imaging, San Francisco, CA, USA). Markers on the scale bars were placed manually on photos if auto detection failed. 

**Figure 2:**
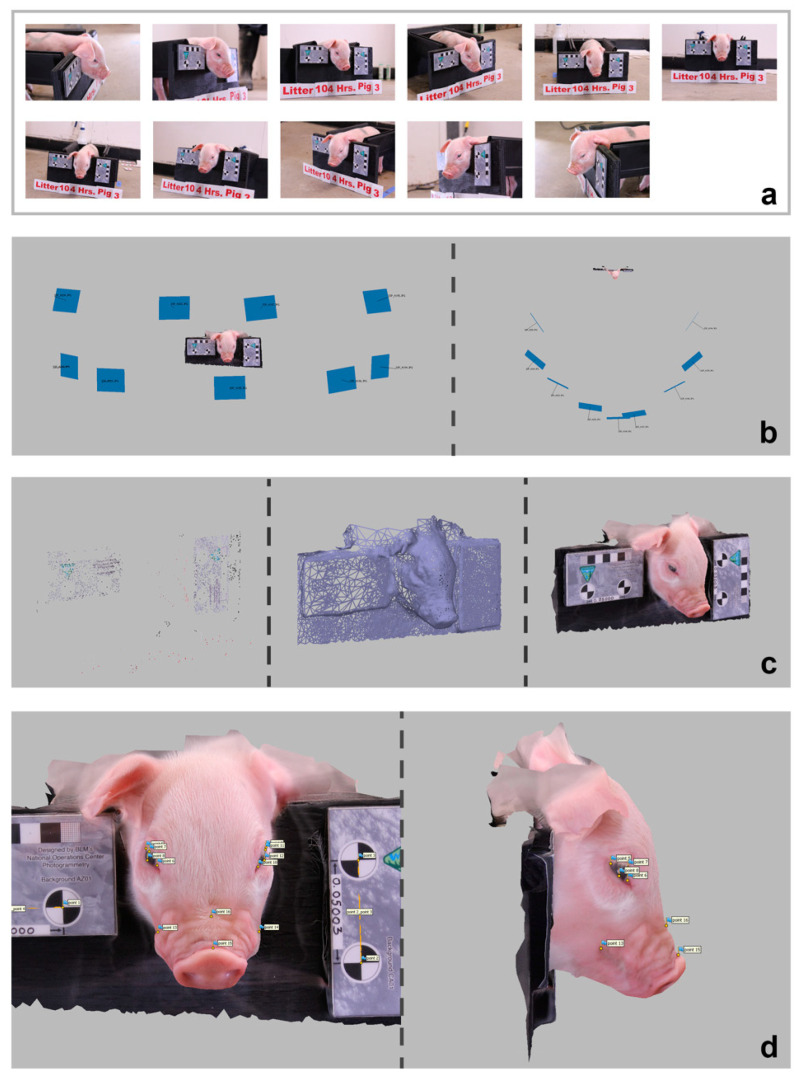
Model Processing in Agisoft Metashape (pig in Litter 10 at 4 h post-castration as example): (**a**) Set of 11 images input into Metashape; (**b**) Set of 9 images successfully aligned in 3D space by Metashape as represented by blue rectangles viewed from the front and above; (**c**) From left to right a sparse point cloud, wireframe, and textured (i.e., with 3D photo overlay) model view of a processed piglet model; (**d**) Location of the 16 landmarks, including 4 landmarks for scale on the cradle.

Landmarks corresponding to three PGS FAUs (orbital tightening, lip contraction, and nose bulge/cheek tension) were then placed on the models using the Markers feature in Metashape. These three FAUs were expected to have the largest detectable range in facial shape change given the quality of the 3D models produced. Landmarks corresponding to ear position and temporal tension were not used, as ears were often missing from the processed Metashape models due to their position relative to the cameras. Temporal tension lacked sufficient visual appearance and was not used. A total of 16 facial landmarks (Table S2) were allocated, with 12 representing the three FAUs as done in previous work [29]. The first four landmarks (1–4) corresponding to the end points of the scale bars were used to generate scale in the models. Landmarks 5–16 were placed manually on the textured view of the 3D models using the Marker tool in Metashape (Figure 2).

### 2.5. The Piglet Grimace Scale (PGS)

#### 2.5.1. PGS 2D Image Analysis

A total of 3872 piglet facial 2D images were taken in this study. From those images, one image of each piglet at each time point was selected for PGS scoring based on optimal quality, angle (i.e., front-profile only), and lighting in accordance with previous work [11,12]. Images that were not in focus or of poor quality were excluded, leaving a total of 352 images (C = 172, S = 180) for final PGS scoring. The researcher selecting the images was blinded to treatment. Each image was assigned a random number generated by Microsoft Excel (Version 16.27; Microsoft Corporation, Washington, DC, USA) to randomize the order in which they were scored to ensure all raters were blinded to treatment and timepoint.

#### 2.5.2. PGS Scoring

**Figure 3:**
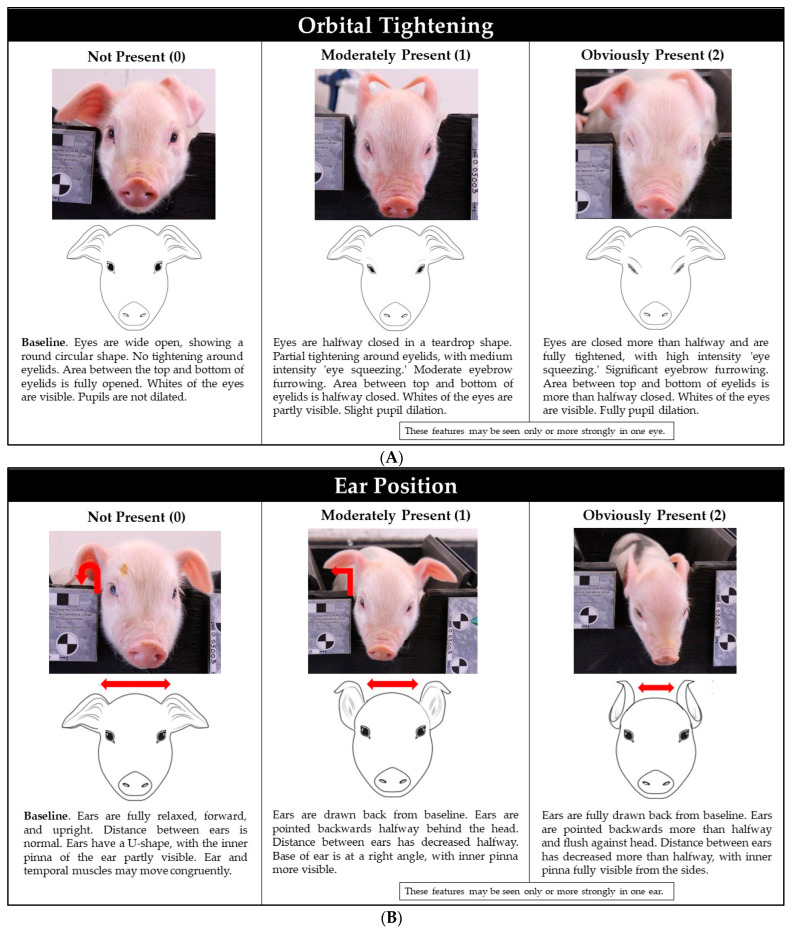

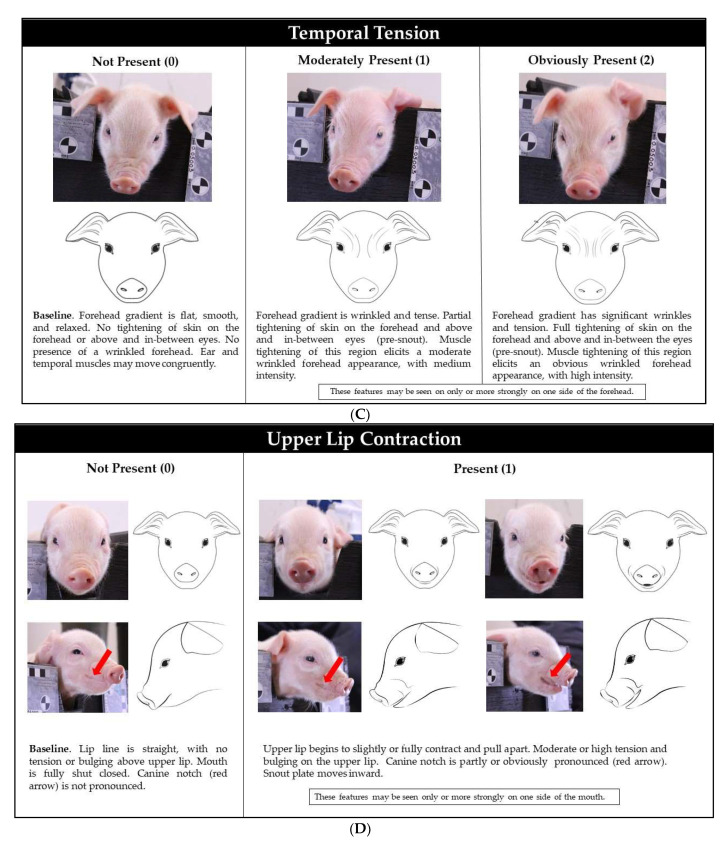

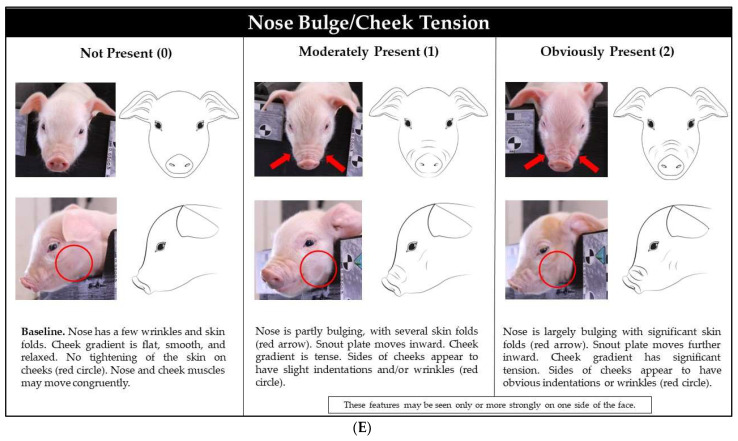
The Piglet Grimace Scale, a pictorial diagram of each facial action unit. Each facial action unit except lip contraction was scored on a 3-point scale (0 = Not Present, 1 = Moderately Present, and 2 = Obviously Present). Lip contraction was scored on a 2-point scale (0 = Not Present and 1 = Present). Grimace sketches were designed by Richard, C. (2020). Orbital tightening, ear position, temporal tension, lip contraction, and nose bulge/cheek tension digital illustrations. Digital Content Library, College of Liberal Arts, University of Minnesota.

The following five FAUs adapted from previous PGS studies were used for scoring: orbital tightening, ear position, temporal tension, lip contraction, and nose bulge/cheek tension [10,11,12]. Each FAU was scored using a 3-point scale (0 = Not Present, 1 = Moderately Present, and 2 = Obviously Present), except lip contraction which used a 2-point scale (0 = Not Present and 1 = Present). From the 352 images, 120 were selected to pre-test the PGS scoring system using the same randomizing method as described above. For the pre-test, four raters with extensive swine backgrounds were chosen to score the 120 images. Prior to the pre-test, raters received a 2 h formal training, which included instruction on all five FAUs, scoring guidelines, and a practice scoring session of sample images. Raters were then given a PGS Pictorial Guide (Figure 3), a score sheet, and the set of 120 images for scoring. Raters were asked to score all 120 images in one sitting to maintain consistent scoring. Scores from the 4 raters were used to calculate the interclass correlation coefficient (ICC) to evaluate inter-rater reliability. After the pre-test, all 352 images were scored using all FAUs by one of the pre-test raters to avoid inter-rater variation.

### 2.6. Statistical Analysis

All data were analyzed using SAS software (Version 9.4; SAS Inst. Inc., Cary, NC, USA). The interclass correlation coefficients (ICCs) for PGS scores were calculated using the Mixed Procedure. The effect of castration treatment on FAUs was analyzed using the Proc Genmod Procedure for non-parametric variables. Dependent variables were grimace scores for each FAU. Treatment and time points were fixed effects, with piglet as the subject for repeated measures. Sham-castration (control) and time point one (T1—baseline) were used as references to castration treatment and other time points, respectively, in the model. Statistical significance was set at odds ratios with 95% confidence intervals differing from 1. Descriptive data were summarized using the Proc Frequency Procedure.

Geometric morphometrics data were first superimposed by Generalized Procrustes Analysis (GPA) using Morpheus et al. Software (Java Edition; Florida State University, Tallahassee, FL, USA). A GPA is designed to eliminate variation unrelated to shape by deleting differences between the models’ position, orientation, and size [32]. Missing landmarks were estimated via GPA mean substitution. The Proc GLIMMIX Procedure for multivariate analysis of variance (MANOVA) was used to test for differences between treatment and time points. Least-square means were compared with the Tukey–Kramer test. Lastly, a Discriminant Function analysis using the cross-validation procedure was used to predict group classification. These analyses were performed on landmarks 5–16 (Table S2; incorporating all FAUs) to determine if there were any differences in shape between castration and sham-castration and among the time points. The landmarks only associated with orbital tightening (landmarks 5–12; Table S2) were run again in separate analyses as this FAU has previously shown to be a more reliable indicator of pain in comparison to the other FAUs [19]. Statistical significance was set at *p* ≤ 0.05. Principal component analyses were performed to visualize the data.

## 3. Results

### 3.1. Inter-Rater Reliability for Facial Action Units

The ICC was highest for orbital tightening (0.68) and ear position (0.67), intermediate for nose bulge/cheek tension (0.54), and lowest for temporal tension (0.40) and lip contraction (0.28).

### 3.2. Castration Treatment Effect on Facial Action Unit

Across the assessment period (T1 to T4), the odds of castrated piglets scoring in a higher grimace category compared to sham-castrated piglets for orbital tightening, ear position, temporal tension, lip contraction, and nose bulge/cheek tension did not differ from 1 (Table 1), suggesting no treatment effect on PGS.

### 3.3. Variation in Facial Action Units over Time

**Figure 4:**
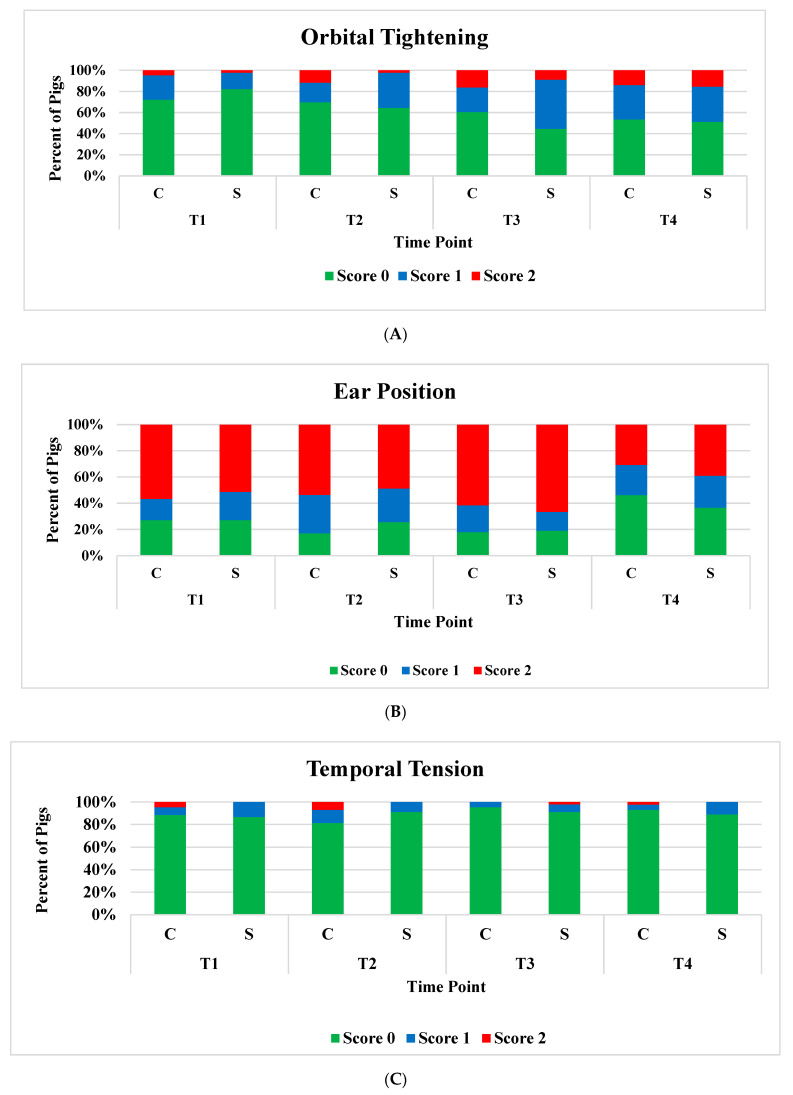

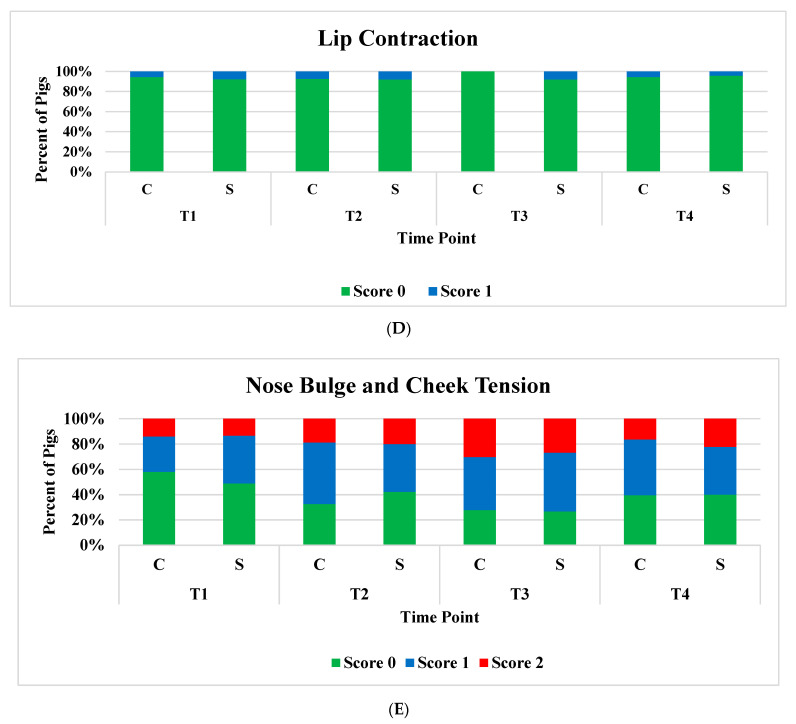
Distribution of (**A**) orbital tightening, (**B**) ear position, (**C**) temporal tension, (**D**) lip contraction, and (**E**) nose bulging/cheek tension scores at each time point (T1 = immediately before castration; T2 = immediately after castration; T3 = 4 h post-castration; and T4 = 22 h post-castration) for both treatments (C = Castration and S = Sham-castration).

The descriptive analysis depicts the percentage of the five FAU scores at each time point for both treatments (Figure 4). Overall, for orbital tightening, about 70% to 80% of piglets scored 0 at T1 (Figure 4A), while approximately 50% of piglets scored 0 at T4. Both treatment groups included all three score types (0, 1, and 2) for orbital tightening at each time point. Over 50% of piglets scored 2 for ear position at T1 (Figure 4B). Less than 30% of piglets from T1 to T3 and about 40% of piglets at T4 scored 0. For temporal tension, more than 80% of piglets scored 0 across all time points and both treatments (Figure 4C). Five percent and 7% of castrated piglets scored 2 at T1 and T2, respectively. Sham-castrated piglets did not score 2 at any time point except T3, where castrated only scored 0 and 1. Given its 2-point scale, lip contraction had the least variation in scores among the five FAUs (Figure 4D); and more than 90% of piglets scored 0 for both treatment groups across all time points. For nose bulge/cheek tension, both treatment groups included all three score types (score 0, 1, and 2) at each time point (Figure 4E).

### 3.4. Application of 3D Landmark-Based Geometric Morphometrics

The principal component analysis (PCA) did not detect any structure, indicating no differences in shape within the data. While differences between treatments were evident across all time points (Wilk’s lambda = 0.8319, F(_33,317_) = 1.94, *p* = 0.002), differences between treatments at individual time points were not detected (*p* = 0.22 for T1, *p* = 0.26 for T2, *p* = 0.96 for T3, and *p* = 0.92 for T4). Differences within time points alone were also not found (*p* = 0.45). The discriminant analysis likewise found no differences between the treatment groups or time points. Using the more conservative cross-validation classification summary (Table 2), castrated piglets were correctly classified at 60.0% and sham-castrated piglets were correctly classified at 53.3%. Correct classification for each time point was 25.0% for T1, 18.2% for T2, 33.0% for T3, and 27.3% for T4. The low percentage scores in the discriminant analysis along with the significant Wilk’s lambda *p*-values indicate that neither the treatment nor the timepoints had any effect on the facial shape.

Similar results were found when rerunning the above analyses for orbital tightening separately. Visualizing the data through PCAs found no discernable structure within the data. No significant differences between treatment by time point or individual time points for orbital tightening were found. In the discriminant function analysis, orbital tightening alone performed better than the entire dataset when comparing treatment groups (castrated piglets were correctly classified at 59.3% and sham-castrated piglets were correctly classified at 61.1%; Table 3). However, in discriminating between treatments and time points, the discriminant function analysis could not confidently discern shape differences (Table 3).

## 4. Discussion

The first objective of the current study was to evaluate the utility of the PGS to detect acute pain. An important consideration made by earlier grimace scale studies was the need to establish a behavioral baseline for the PGS [9]. To improve the reliability of facial expressions as a method of pain assessment, a clear difference between a positive control (i.e., castration without a pain agent) and negative control (i.e., sham-castration) is necessary to compare responses influenced by pain mitigation methods such as analgesics and anesthetics [10]. Thus, this study focused on surgical castration and sham castration alone, a key difference from previous PGS studies [10,11,12]. A second key difference was the upgraded format of our pictorial guide to improving inter-rater reliability, which unlike previous PGS studies included a real-time photo, sketch, and description for each individual score to help raters understand what specific features should be observed as the severity of pain increases from score 0 to 2. In conjunction with the pictorial guide, our raters received formal training. We observed that orbital tightening, ear position, and nose bulge/cheek tension may be useful pain indicators based on inter-rater reliability. However, differences between treatments were not detected by the PGS nor 3D landmark-based geometric morphometrics.

### 4.1. Inter-Rater Reliability

Inter-rater reliability among the four raters in this study was an essential measure of PGS utility as a measure of scoring consistency. According to Koo and Li [33], the ICCs among the four raters in the current study for orbital tightening (0.68), ear position (0.67), temporal tension (0.40), lip contraction (0.28), and nose bulge/cheek tension (0.54) were indicative of moderate, moderate, poor, poor, and moderate inter-rater reliability, respectively. Our ICC results for orbital tightening were promising but lower than other PGS studies. Gottardo et al. [10] and Di Giminiani et al. [11] observed an ICC of 0.83 among 3 raters and 0.95 among 30 raters for orbital tightening, respectively. Our ICC results for both orbital tightening and ear position align with other grimace scales for mice, rats, rabbits, cats, horses, and cows [1,4,5,6,34,35]. Tightening of the eyes is an evolutionary conserved facial expression acting as a protective mechanism, concealing sensitive areas of the face from a threat [2]. Backward facing or flattened ears are commonly expressed during aversive experiences [2]. As prominent features of the face, it is, therefore, reasonable to presume that both the eyes and ears may be easier to recognize when attempting to score facial expressions.

Temporal tension was an FAU adapted from Di Giminiani et al. [11], who demonstrated excellent inter-rater reliability for this FAU. However, Di Giminiani et al. [11] enrolled both male and female piglets undergoing two treatment procedures (i.e., castrated males and tail docked females), where females underwent teeth clipping prior to tail-docking and males were restrained while photographs were taken [11]. It is possible that Di Giminiani et al.’s study procedures may have induced greater activation of temporal muscles given that females were exposed to two painful procedures, and males were exposed to further handling [11]. Moreover, temporal muscles move congruently with the ears and simultaneous involuntary muscle movement in both locations is likely to increase with additional handling and surgical procedures [2]. Grimace differences influenced by sex may have also occurred.

Temporal tension may be a particularly challenging FAU to score in some species. A study establishing the Horse Grimace Scale (HGS) reported difficulties with 21% of their images in scoring an FAU termed “tension above the eyes” although scoring of the remaining images resulted in an ICC of 0.86 [34]. In that study [34], castrated stallions were of mixed breed, age, and color, while horses in the control group were of mixed age and sex, with both groups receiving a non-steroidal anti-inflammatory drug (flunixin meglumine). Biological differences or analgesic effects alone could have facilitated the visibility and scoring of FAUs. In contrast, the set of images in this present study only compared castrated and sham-castrated piglets not given any form of pain management. We also note that landmarks could not be added to denote temporal tension on the 3D models in our current study. It is possible that our study procedures failed to activate temporal muscles in response to castration, or that the fairly light-colored skin of piglets made temporal tension harder to detect without sufficient textured appearance [24]. Lip contraction was also adapted from previous work in piglets undergoing tail docking and castration [11], where it was ultimately excluded as it could not be scored in 30% of images. In our study, lip contraction was scored on a 2-point scale as it was less visible in front profile images; however, this FAU received the lowest ICC and could not be scored in 40% of our images Costa et al. [34] reported similar results on horse FAUs associated with the mouth and lips (i.e., “prominent strained chewing muscles” and “mouth strained and pronounced chin”), as visibility of muscle activation in the mouth/jaw region are more prominent in side-profile images. Another reported challenge of scoring FAUs in the mouth and jaw region is the misinterpretation of their causation (e.g., by pain-activated muscle movement or communicative vocalizations) [2]. Lastly, changes in the nose and cheeks have been evaluated separately in mice [1], rabbits [5], and horses [34], while in rats [4] have been combined into a single FAU. Species specific differences may cause the nose and cheeks to either flatten or tense up, which need to be considered in FAU development. In piglets, nose bulging and cheek tension specifically have been examined alone [10,11] and together [12]. Independently, cheek tension reliability was found to be both poor [10] and good [11], with nose bulging being excellent [11]. In the present study, nose bulge and cheek tension were combined as nose and cheek muscles were observed congruently contracting in piglets, with results showing moderate inter-rater reliability. Our results aligned well with others who also scored these two FAUs as one in piglets [12].

### 4.2. Castration Treatment Impacts

Facial expressions can differ in terms of how they are expressed (e.g., their duration or intensity) [2]. Facial expressions in relation to pain should be seen with continuous or more frequent observation and may be difficult for still images to detect [2]. In our study, the probability of castrated piglets scoring in a higher grimace category (i.e., 1 and 2 vs. 0) did not differ from sham-castrated piglets for all five FAUs across all time points. Comparably, Gottardo et al. [10] reported no significant differences between castrated and sham-castrated piglets for orbital tightening. In contrast, Di Giminiani et al. [11] reported a change between pre-and-post-castration scores for orbital tightening in tail-docked piglets, while Viscardi et al. [12] observed higher PGS scores at 0, 3, 4, and 5 h post-castration. While not immediately clear as to why treatment effects were not observed in the present study as previously reported, piglets in Di Giminiani et al. [11] were also exposed to teeth clipping prior to castration, a procedure known to elicit pain [36]. Similarly, Viscardi et al. [12] exposed piglets to tail-docking and also provided pain management (meloxicam and eutectic mixture of local anesthetic (EMLA) cream), where it is possible that those additional factors may have accounted for the differences observed [12].

### 4.3. Applying Photogrammetry and 3D Landmark-Based Geometric Morphometrics to the PGS

To the best of our knowledge, this is the first study to analyze changes in facial shape in piglets through the application of photogrammetry and 3D landmark-based geometric morphometrics. We hypothesized that the application of these technologies would refine PGS data collection and analysis by producing higher quality images and minimizing observer bias. In the present study, photogrammetry was indeed successful in obtaining clear, high-resolution images. An added benefit of using a photogrammetry rig is taking multiple simultaneous images, which is a critical component for photogrammetry of live, moving objects. This may help in capturing the nature of pain-related facial expressions, as they should be seen from different angles, then a single frame can detect them. However, suspected scoring difficulties in this study were related in part based to certain FAUs being visible in a front-profile view (e.g., orbital tightening) while others were best observed from the side (e.g., lip contraction), an issue in which the photogrammetry rig was unable to improve.

Application of geometric morphometrics in combination with photogrammetry also showed success in producing 3D models of good quality in the current study. A key construct of geometric morphometric analysis is that all landmarks must be placed in the same location in each model. In our study, parts of piglet faces could not be fully reconstructed in multiple models given insufficient pixel data. The eyes were the only FAU fully reconstructed in all 3D models in this study. The 3D models provided the ability to annotate each eye with four landmarks. These four landmarks were placed in specific areas to capture the full range of motion when tightening. Lips and noses were moderately reconstructed, while the ears and forehead failed reconstruction for most models. Thus, only the corners of the lips and the two prominent wrinkles of the nose were annotated. As a result, analysis of orbital tightening was possible in comparison to all other FAUs.

Furthermore, the construction of high quality 3D models requires photogrammetry images to have sufficient features and textures [24,25], which natural piglet surface morphology may lack. Future work can improve models by incorporating more cameras, especially in areas above and below the subject providing a full 360° view. Furthermore, if a restraining device is used, then placing the rig above the ground could help capture all necessary angles. Facial shape differences between treatments across all time points were detected, although when timepoints were analyzed separately no differences were found. The observed facial shape differences may have been due to natural individual variations among piglet faces. Discriminant analysis also did not detect any distinct facial shape differences as piglets were correctly classified roughly 50% and 25% of the time for each treatment and timepoint, respectively. A classification of 70% or greater is considered ideal representing group differences beyond biological variation. Thus, the application of 3D landmark-based geometric morphometrics was not able to quantify changes in the facial shape of piglets, at least under the conditions of the current study. 

Measuring distances between landmarks in 2D landmark-based geometric morphometrics have been used to develop a Feline Grimace Scale (FGS) [30,35]. Images of 2D from video recordings were annotated for both FGS studies [30,35]. Evangelista et al. [35] found significant differences between treatment groups (healthy cats vs. cats with abdominal pain) in terms of linear distance ratios and angles in relation to five FAUs (orbital tightening, ear position, muzzle tension, whiskers position, and head position). However, the cats used in that study were a combination of seven different breeds and the causes of abdominal pain (e.g., lymphoma, inflammatory bowel disease, and pancreatitis) were inconsistent [35]. Finka et al. [30] was able to quantify changes in facial expression through principal component analysis, indicating distinct shape variation for the ears, muzzle, cheeks, and eyes using cats of mixed breed undergoing an ovariohysterectomy. They found that T2 (1 h post-surgery) was associated with the largest intensity of pain, but anesthesia administration prior to T2 should be considered [30]. Although both studies were able to detect changes in facial shape in relation to pain, differences in breed, coat color, types of painful stimuli, and drug effects may be confounding and should be controlled for in the experiment design [9,34]. Additionally, the use of 2D images warrants caution in not retaining the geometric integrity of landmarks as in 3D models, where distances and angles are unaffected. Finka et al. [30] used both 2D front and lateral images and such factors can be misinterpreted. Using the 3D model, Cappai et al. [25] found no differences between in vivo and virtual bull body measurements. The 3D models predicted wither’s height, body length, and rear trimness of bulls with an 89% positive predictive value and 100% accuracy. However, only two bulls were used in their study. Future research with a large number of animals of each species will be needed before 3D imaging can be applied on-farm for the evaluation of livestock morphology. 

Our study may have also been limited by certain factors. Evidence indicates handling and manipulation alone cause stress and discomfort in piglets, amplifying facial expressions [37]. Additionally, novel items (e.g., the piglet cradle) can cause animals to experience neophobia [38], so it is possible that stress induced by our study procedures may have prevented discrimination of facial differences induced by castration pain. Future studies should habituate pigs to any new restraint devices and measuring equipment. Moreover, piglets in the current study were castrated within 24 h after birth as necessitated by existing on-farm research protocols. Pigs of an older age may have better suited for this study, as newly born pigs have wet ears and excessive face wrinkling that would otherwise settle with time. Within the first 24 h of life, pigs have rapid morphological changes and need time to acclimate to their environment [11]. Previous PGS studies began data collection at an age range of 4 to 5 days, which may have facilitated scoring through more prominent FAU visibility [10,11,12].

## 5. Conclusions

Our findings suggest that facial action units of orbital tightening, ear position, and nose bulge/cheek tension may be useful indicators of the Piglet Grimace Scale (PGS) given the inter-rater reliabilities achieved in this study. Furthermore, the application of photogrammetry and 3D landmark-based geometric morphometrics improved the quality of data collection and analysis in producing high-resolution images and 3D models of good quality for PGS scoring and adequate landmark annotation, respectively. Further refinements are necessary to improve the sensitivity of these pain assessment methods and technologies to detect pain-related changes in piglet facial expressions and shapes.

## Figures and Tables

**Table 1:** Likelihood of castrated piglets scoring in a higher grimace category compared to sham-castrated piglets ^1^.

FAU ^2^	Odds Ratio ^3^	95% CI_L_^4^	*p*-Value for Odds Ratio
Orbital Tightening	0.95	0.50	0.87
Ear Position	0.97	0.50	0.93
Temporal Tension	1.03	0.38	0.95
Lip Contraction	0.66	0.17	0.54
Nose Bulge/Cheek Tension	0.98	0.52	0.95

^1^—Proc Genmod was used to analyze the effect of treatment (castration vs. sham-castration) across all time points (T1 to T4) with treatment and time point as fixed effects and piglet as the subject for repeated measures. T1 = immediately before castration; T2 = immediately after castration; T3 = 4 h post-castration; and T4 = 22 h post-castration. ^2^—Facial action units. ^3^—Odds of castrated pigs scoring in a higher grimace category. ^4^—Lower limit of 95% confidence interval (CI) for odds ratios. Odds ratios with 95% CI differ from 1 were considered significant.

**Table 2:** Cross-validation discriminant analysis results utilizing all shape landmarks ^1^.

**Treatment**	
Castration	60.0%
Sham-Castration	53.3%
**Time Point ^2^**	
T1	25.0%
T2	18.2%
T3	33.0%
T4	27.3%

^1^ Results represent the percent of piglets correctly classified. ^2^ T1 = immediately before castration; T2 = immediately after castration; T3 = 4 h post-castration; and T4 = 22 h post-castration.

**Table 3:** Cross-validation discriminant analysis results utilizing landmarks capturing orbital tightening ^1^.

**Treatment**	
Castration	59.3%
Sham-Castration	61.1%
**Time point ^2^**	
T1	25.0%
T2	20.5%
T3	27.3%
T4	31.8%

^1^ Results represent the percent of piglets correctly classified. ^2^ T1 = immediately before castration; T2 = immediately after castration; T3 = 4 h post-castration; and T4 = 22 h post-castration.

## Data Availability

Raw data supporting the results of this article will be made available by the authors upon request according to the University of Minnesota Institutional Animal Care and Use Committee (IACUC) Policy: Photography, Video and Audio Recording of Animals Used in Research and Teaching.

## Supplementary Materials

The following supporting information can be downloaded at: https://www.mdpi.com/article/10.3390/ani12151944/s1, Table S1. Settings used to process images into textured 3D models in Agisoft Metashape; Table S2. Location of landmarks placed on the 3D piglet models.
