# Supplementary material for: The Application of 3D Landmark-Based Geometric Morphometrics towards Refinement of the Piglet Grimace Scale
Source: Animals (Basel). 2022 Jul 30;12(15):1944. doi: 10.3390/ani12151944 (PMC9367447; doi:10.3390/ani12151944)
Supplement: Supplementary file 1 [file animals-12-01944-s001.zip › animals-1818141-supplementary.pdf]

Table S1. Settings used to process images into textured 3D models in Agisoft Metashape

**Image Alignment**	
Accuracy	High
Key Point Limit	75,000
Tie Point Limit	75,000
Adaptive Camera Model Fitting	Yes
**Build 3D Model**	
Source Data	Depth Maps
Surface Type	Arbitrary
Face Count	250,000
Interpolation	Enabled
Calculate Vertex Colors	Yes
**Build Texture**	
Mapping Mode	Generic
Blending Mode	Mosaic
Texture Count / Size	1 × 4096 pixels
Hole Filling	Yes
Ghost Filter	Yes

Table S2. Location of landmarks placed on the 3D piglet models

Marker	Position
1-4	End points of scale bars
5	Outer corner of right eye
6	Inner corner of right eye
7	Top of right eye
8	Bottom of right eye
9	Outer corner of left eye
10	Inner corner of left eye
11	Top of left eye
12	Bottom of left eye
13	Corner of right lip
14	Corner of left lip
15	Tip of the snout (superior point of the end of the snout)
16	Base of the snout (center of proximal nose wrinkle)
